# The Use of Acrylate Polymers in Dentistry

**DOI:** 10.3390/polym14214511

**Published:** 2022-10-25

**Authors:** Milena Kostić, Marko Igić, Nikola Gligorijević, Vesna Nikolić, Nenad Stošić, Ljubiša Nikolić

**Affiliations:** 1 Department of Prosthodontics, Faculty of Medicine, University of Niš, 18000 Niš, Serbia; 2 Faculty of Technology, University of Niš, 16000 Leskovac, Serbia; 3 Department of Restorative Dentistry and Endodontics, Faculty of Medicine, University of Niš, 18000 Niš, Serbia

**Keywords:** acrylic polymers, dentistry, biocompatibility

## Abstract

The manuscript aimed to review the types of acrylate polymers used in dentistry, as well as their chemical, physical, mechanical, and biological properties. Regarding their consistency and purpose, dental acrylate polymers are divided into hard (brittle), which includes acrylates for the production of plate denture bases, obturator prostheses, epitheses and maxillofacial prostheses, their repairs and lining, and soft (flexible), which are used for lining denture bases in special indications. Concerning the composition and method of polymerization initiation, polymers for the production of denture bases are divided into four types: heat-, cold-, light-, and microwave-polymerized. CAD/CAM acrylate dentures are made from factory blocks of dental acrylates and show optimal mechanical and physical properties, undoubtedly better monomer polymerization and thus biocompatibility, and stability of the shape and colour of the base and dentures. Regardless of the number of advantages that these polymers have to offer, they also exhibit certain disadvantages. Technological development enables the enhancement of all acrylate properties to respond better to the demands of the profession. Special attention should be paid to improving the biological characteristics of acrylate polymers, due to reported adverse reactions of patients and dental staff to potentially toxic substances released during their preparation and use.

## 1. Introduction

In an effort to eliminate the functional and aesthetic defects caused by natural tooth loss, various types of materials were used for the production of dental restorations. Production of synthetic polymers began at the end of the nineteenth and the beginning of the twentieth century, making it a revolutionary discovery in biotechnology. Already in the 1940s, poly (methyl methacrylate) (PMMA) became the material of choice for about 60% of mobile dental prostheses made on the American market. Even though they were introduced into dentistry almost a century ago, acrylate polymers are still considered irreplaceable in the field of their application. Acrylate polymers are transparent materials that can easily be modelled and painted, and that faithfully imitate lost teeth, gingiva, and skin [1].

Acrylate polymers owe their wide use in dentistry to their optimal mechanical and physical properties (strength, hardness, dimensional stability, resistance to abrasion, low solubility and water absorption), chemical stability, satisfactory biological acceptability, aesthetic characteristics, long-lasting colour, absence of odour and taste, simple processing and repair, and cheap price [2,3].

Regardless of the number of advantages that these polymers have, they also exhibit certain disadvantages like fracture of dentures due to water sorption and poor impact and flexural strength [4]. Technological development makes it possible to enhance all acrylate properties to respond better to the demands of the profession. Special attention should be paid to improving the biological characteristics of acrylate polymers, due to reported adverse reactions of patients and dental staff to potentially toxic substances released during their preparation and use [5]. The addition of biocidal agents to materials in contact with tissues can improve their biological properties and reduce the possibility of infection in the patient’s mouth [6,7,8]. This manuscript aims to describe the characteristics of acrylate polymers that have multiple purposes in dental practice, as well as the possibility of their improvement in terms of mechanical and physical characteristics and improvement of their biological value. 

References used in for this research were systematically hand-searched in four academic literature databases: MEDLINE/PubMed, Web of Science, Scopus and Google Scholar using keywords specific to the subtitle in the manuscript, regardless of the year of publication.

The aim of the manuscript was to review the types of dental acrylate polymers, as well as their chemical, physical, mechanical, and biological properties.

## 2. Use of Acrylate Polymers in Dentistry

Acrylate polymers represent one of the four most commonly used groups of materials in modern dentistry, primarily in prosthodontics. As building materials, acrylates are used for the production of the base plate of mobile dentures, artificial teeth, temporary crowns and bridges, mobile orthodontic appliances, obturators and maxillofacial prostheses, as well as for the needs of lining and repairing these restorations. As auxiliary materials, acrylates are used for individual tray production, models of prosthetic restorations, bite blocks, and less often for the production of occlusal ridges. Special types of acrylates are an integral part of composite and modified glass ionomer cements [9].

## 3. Types of Acrylate Polymers in Dentistry

According to their consistency and purpose, dental acrylate polymers are divided into hard (brittle), which includes acrylates for the production of denture plate bases, obturator prostheses, epitheses and maxillofacial prostheses, their repair and lining, and soft (flexible), which are used for lining denture bases in special indications: conditioning of oral tissues, cushioning of masticatory pressure on the mucous membrane of the denture support, and treatment of inflammation [10].

Regarding the composition and the method of polymerization initiation, polymers for the production of denture bases are divided into four types (ISO 1567: 1999): heat-, cold-, light-, and microwave-polymerized [11]. 

## 4. Composition of Dental Acrylate Polymers

According to their composition, acrylate materials are esters of poly(acrylic) acid. PMMA (IUPAC name: poly [1-(methoxy carbonyl)-1-methyl ethylene]) is a synthetic polymer prepared by the free radical addition and polymerization of methyl methacrylate (C_5_O_2_H_8_) to poly methyl methacrylate (C_5_O_2_H_8_)n [12]. The polymerization reaction is initiated by the creation of free radicals chemically or by heat, microwave or light energy. The propagation phase involves the binding of monomers, followed by the termination phase and the completion of polymer chains by the reaction of monomers and free radicals [4].

PMMA is conventionally available in the form of a powder–liquid system. The composition of hard acrylate materials used for the manufacture of denture bases is given in Table 1 [10,13].

The most commonly used heat-polymerized dental acrylates are used to make denture bases. Polymerization occurs by mixing the powder and liquid components (Table 1) in the presence of thermal energy (water bath), which activates the polymerization initiator (dibenzoyl peroxide). Dibenzoyl peroxide dissociates into carbon dioxide creating free radicals at a temperature of 70 to 100 °C. Conventional heat curing cycle involves a longer curing time (~9 h at 74 °C); and today often used rapid-heat-polymerized PMMA requires a significantly shorter (20 min at 100 °C) curing time [14].

The polymerization process can also be started with microwave energy, and the main advantage of this preparation is the time efficiency (three minutes at 500 w) in the microwave, which results in compensations with similar mechanical physical properties as conventional mechanical heating. These materials do not contain benzoyl peroxide as a polymerization initiator [15]. 

Denture lining is indicated in case of readaptation of the denture after resorption or atrophy of the oral tissues supporting the denture. Materials that are the same or very similar in physical and chemical characteristics to the materials from which denture bases are made are used during this process. Denture lining can be performed directly in the patient’s mouth by a standard clinical procedure, using PMMA-based material (hard acrylate) or poly (ethyl methacrylate)-based material (PEMA) soft (resilient) acrylate to act as an oral conditioner to prevent inflammation by reducing mechanical irritation or damping of forces acting on the alveolar ridge [16].

Cold polymerized PMMA (chemically polymerized or auto polymerizing PMMA) contains a polymerization accelerator (N,N dimethyl-p-toluidine, tertiary amines, sulfuric acid) that activates benzyl peroxide, creating free radicals to initiate polymerization [4]. The degree of polymerization of cold polymerized acrylates compared to hot polymerized ones is significantly lower, and the amount of residual monomer affects the biological, physical and mechanical properties of the acrylate material [1].

Regarding soft dental acrylates, the polymer is most often PEMA, but it can also be PMMA, as well as PBuMA. Liquids include ester plasticizers (dibutyl phthalate, butyl glycolate) in a percentage of 30% to 60% and ethanol as a solvent, in a proportion of 4–60% of the total mass. Plasticizers provide softness or resilience to this type of material at body temperature. Plasticizers are soluble in oral fluids, and their gradual loss over time leads to hardening of the material in the patient’s mouth [17]. Given the proven toxicity of phthalates, the most commonly used plasticizers in dental acrylates, their replacement with citrates would significantly improve the biological value of dental acrylates. The characteristic of soft acrylates is that they change their composition and consistency over time and harden during their function in the orofacial system, which is in accordance with the indications assigned to them [18].

Unlike classic two-component systems, light-polymerized acrylates are single-component plates or gels, which are easily adapted to the working model and are only then polymerized. According to their composition, they are urethane dimethacrylate (UDMA) with micro silica filler [19]. The material has found application in the repair of dentures and the production of individual trays [19].

## 5. Physico-Mechanical Properties of Dental Acrylate Polymers

All dental building materials become an integral part of the orofacial system and are exposed to the action of high-intensity masticatory forces. Therefore, they must possess optimal mechanical and physical properties.

To meet the expectations, dental acrylate polymers need to have the following: supreme hardness, impact strength (restoration fracture resistance), fatigue strength (resistance to stress in functions of the orofacial system), high tensile strength, abrasion resistance, high modulus of elasticity and good resilience [20]. Research done by Kawaguchi et al. has shown that the flexural strength of the specimens with Mw 220,000 and 350,000 PMMA beads was significantly higher than the strength of specimens with beads having other molecular weights [21]. The desired physical characteristics of these acrylates have low specific weight so that the dental restoration is as light as possible, as well as thermal and dimensional stability [22]. 

Differences in these properties among various types of dental acrylates depend on the method of polymerization, as well as the average molecular weight of the polymer. Thus, the presence of residual monomer (MMA) of low molecular weight reduces the hardness, strength, and elasticity modulus of acrylate materials. Even though the hardness, strength, and resistance impact of completely polymerized cross-linked materials are less compared to tooth tissues, acrylates meet the requirements set by the profession. The mechanical properties can be improved by incorporating grains and fibres of glass, zirconium oxide, aluminium oxide, or silicon carbide into a conventional acrylate mass, although there may be a problem with material homogenization [23]. Due to low elasticity, acrylates are brittle and prone to fracture. The fracture resistance of acrylate materials can be improved by incorporating elastomers (vinyl copolymers), or polyethylene, polyester, and nylon fibres with a high modulus of elasticity within their matrix [24].

The physico-mechanical properties of heat-polymerized dental acrylates are better in comparison to cold-polymerized ones, which is explained by their more complete polymerization. The majority of research has shown that microwave-polymerized acrylates have mechanical and physical properties similar to heat-polymerized acrylates [25,26].

On the other hand, single-component light-polymerized acrylates exhibit a more complete bonding, less residual monomer, and therefore better mechanical quality and biocompatibility [27]. 

The use of Computer-Aided Design and Manufacturing (CAD/CAM) for the production of dentures has been on the constant increase. CAD/CAM acrylate dentures are made from factory blocks of dental acrylates and show optimal mechanical and physical properties, undoubtedly better monomer polymerization and thus biocompatibility, and stability of the shape and colour of the base and dental implants [28].

**Figure 1:**
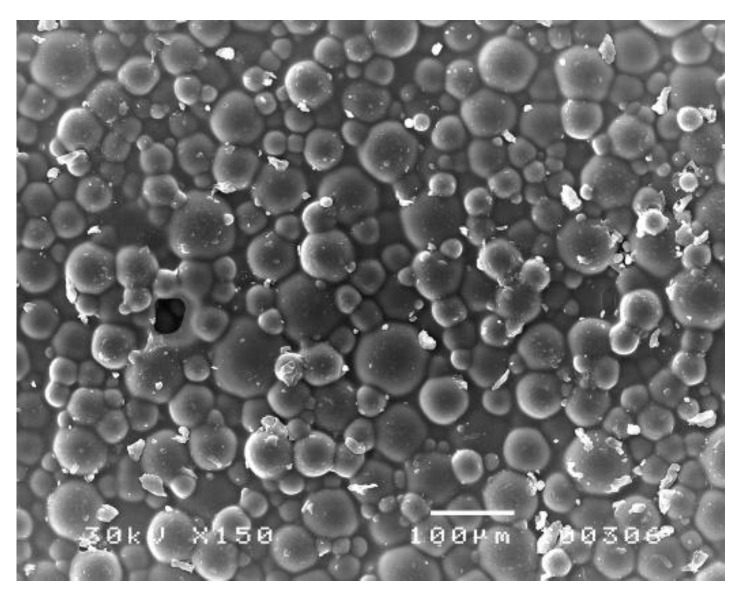
Fused granules of firm cold-polymerized acrylate samples, up to 100 µm in size. The material showed partial porosity [33].

Higher porosity of cold-polymerized acrylates is a consequence of faster polymerization under poorly controlled conditions [13]. Their internal porosity makes them less resistant to fracture, while the surface porosity enables better adherence of biofilm and food residues [29] (Figure 1). Additional polymerization or post-polymerization could improve these properties, as shown by some studies [30,31,32].

Kostić et al. detected a higher concentration of residual monomer in samples of cold- compared to heat-polymerized dental acrylates. However, the post-polymerization treatment in the water bath at 100 °C, in the microwave oven, and by placing in the water bath, eventually led to its reduction. The level of monomer conversion after polymerization of heat-polymerized acrylates did not require additional post-polymerization treatments [34,35].

Incomplete polymerization increases liquid absorption and solubility of dental acrylates. Residual monomer acts as a plasticizer in the material, but the decrease in the quality of the material can be attributed to the release of residual monomer into saliva and the compensatory absorption of liquid from the oral environment, which leads to the plasticization of the material and makes it more flexible and resilient [36]. The amount and rate of water absorption depend on network density, potential hydrogen bonds, and polar interactions. Denser polymer networks have a lower possibility of water absorption [37]. According to ISO 1567, the increase in bulk density of dental acrylate polymers per unit volume (water absorption) must not exceed 32 µg/mm^3^ [11]. Although all dental acrylates show water absorption within the standardized limits, significantly higher values were observed for cold-polymerized ones, which is the consequence of their weaker polymerization [38].

Analogous to water absorption, a more compact dental acrylate material shows lower solubility. The solubility of acrylates in saliva is negligible. According to ISO 1567, the acceptable solubility is 1.6 µg/mm^3^ for heat- and 8 µg/mm^3^ for cold-polymerized dental acrylates [11]. However, one should not disregard the fact that acrylate restorations release residual monomer, benzoyl peroxide, plasticizers, and other substances into the oral environment, which dissolve in saliva and act on oral tissues. Soft acrylate plasticizers are soluble in oral fluids and their predicted loss over time leads to the hardening of the material in the patient’s mouth [39]. 

Dental acrylate polymers show lower abrasion resistance compared to teeth. Consequently, dentures erode easily if they are opposed by natural teeth or fixed restorations [24].

Acrylates are dimensionally stable materials, and possible problems can be expected at the points of connection of dentures and orthodontic appliances with other materials, due to internal stress as a result of differences in thermal conductivity [20].

## 6. Biofilm on the Surface of Dental Acrylate Polymers

The biological activity of dental acrylates can be observed through two aspects: their interaction with tissues and agents from the environment and the release of potentially toxic substances with local and systemic side effects [5,35].

The interaction of acrylate materials with the environment is determined by the surface design of the material, primarily its roughness. The roughness of acrylates depends on the type, i.e., the polymerization procedure, as well as the final treatment or polishing of the dental restoration [40]. The uneven surface of acrylate materials is a preferred place for the accumulation of plaque, pigments, food and drink residues, as well as decayed oral tissue [41]. 

**Figure 2:**
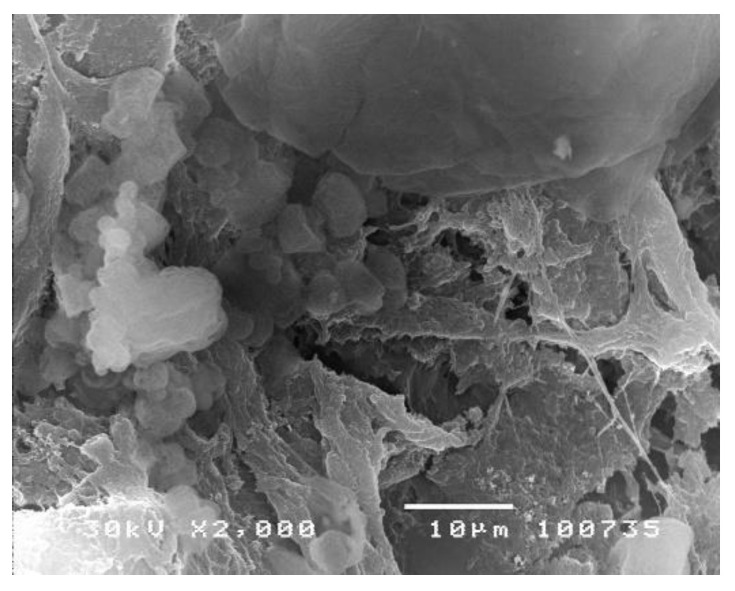
Tissue remains on soft dental acrylate polymer [43].

To compare the adhesive ability of soft and hard dental acrylate polymers, Kostić et al. analysed their structure after a four-week intramuscular implantation by employing SEM analysis (Scanning Electron Microscopy) [42]. The implanted acrylate samples showed a high adhesive potential for the surrounding muscle tissue. After their removal, the tissue remains, with a tendency of growing into the material, could be seen on the surface. Samples obtained by cold polymerization showed a significantly higher degree of adhesion compared to heat-polymerized acrylates (Figure 2).

The obtained results showed that the tissue grows into the porous acrylate material and can decompose in it. Consequently, the polymer becomes the collector of infectious material, enabling recurrent infections. Considering the inhomogeneity of the internal and external structure of dental acrylate polymers, flawless hygiene of dental prostheses is imperative [43]. 

**Figure 3:**
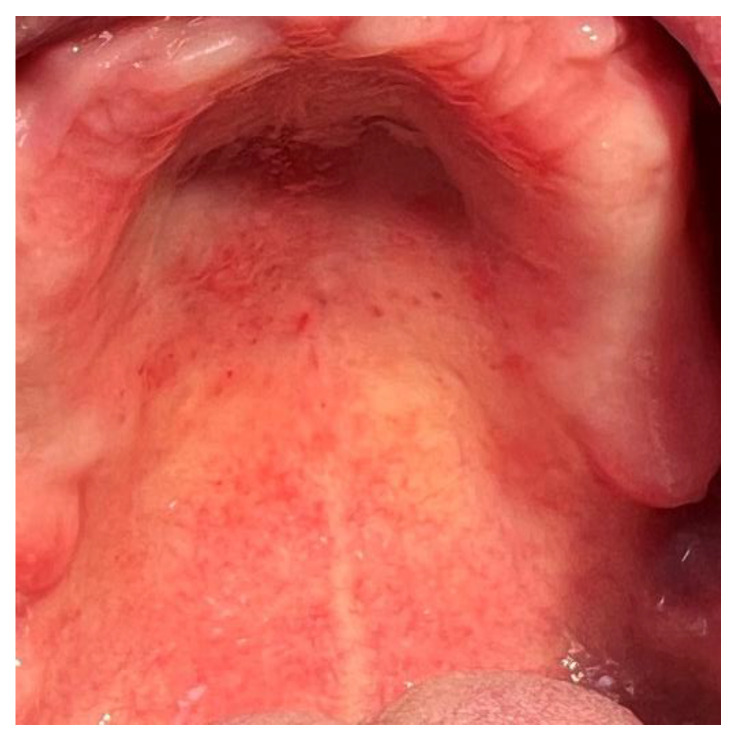
Denture stomatitis in the upper jaw.

The most common recurrent infection associated with wearing acrylate dentures is denture stomatitis (*Stomatitis prosthetica*), which occurs in 70% of wearers of acrylate dentures (Figure 3) [44]. Fungi of the genus *Candida*, especially *Candida albicans*, are mainly responsible for the occurrence of denture stomatitis. It is a dimorphic fungus that is a commensal colonizer of the oral cavity, gastrointestinal and reproductive systems. In immunodeficient states, *C. albicans* may become virulent and cause candidiasis. Surface roughness, salivary pellicle, hydrophobicity, and electrostatic interactions are the predilection factors that affect adhesion and biofilm formation of *Candida* on acrylate dentures [45]. 

*C. albicans* has the ability of a multicellular growth form, and its transition to the mycelium form enables easy adhesion to acrylate material. In the patient’s mouth, acrylate polymer is coated with salivary pellicle formed by the precipitation of salivary mucin and glycoproteins [46]. The surface of acrylate polymers is hydrophobic and Candida binds to it by hydrophobic interactions and electrostatic forces [47,48]. Surface free energy proportionally increases Candida adherence [49,50]. 

Complex biofilm on the surface of acrylate materials can be the cause of other oral infections, but above all the cause of damage to the periodontal tissue and caries on the remaining teeth [51,52]. Cases of aspiration pneumonia and gastrointestinal infections associated with denture stomatitis [53] have also been reported in the literature. To prevent various infections triggered by the presence of biofilm on the surface of acrylate dental polymers, it is necessary to work on its removal and reduction, but also on improving the performance of the material itself.

Candida reproduces easily in the porous and moist environment of acrylate material, thus is often more abundant on dentures themselves than on the oral mucosa [54]. The contamination of dentures is irreversible and prevents healing of the oral cavity. Biofilm surrounded by an extracellular matrix is a biological barrier for the penetration of antifungal drugs. Within the biofilm, phenotypic changes occur as a result of limited availability of nutrients and oxygen, which consequently reduces their sensitivity to antimicrobial drugs [55,56]. 

The incorporation of fluconazole, chlorhexidine, amphotericin B, nystatin, and others improves the antimicrobial properties of acrylate polymers and limits the formation of biofilm on dentures [57,58].

The antimicrobial properties of nanostructured silver, impregnated into acrylate materials for the production of dentures, against *C. albicans* and *Staphylococcus aureus* were also confirmed—in proportion to the concentration used [59,60,61]. Examining the antimicrobial activity of silver incorporation into PMMA, Gligorijević et al. showed the best antimicrobial activity against *C. albicans* (MIC = MMC = 3.13 mg/mL) was identified for samples containing 10% AgCl PMMA, for both cold and heat curing PMMA. Slightly lower activity of these samples was shown against S. aureus (MIC/MMC = 3.13/6.25 mg/mL). 10% AgNPs PMMA of both cold- and heat-curing showed lower activity on the tested microorganisms compared to samples with the 10% AgCl PMMA, with MIC = MMC = 12.50 mg/mL [60]. The adhesion of biofilm was significantly reduced by incorporating zirconium dioxide and titanium dioxide into the acrylate structure [62,63].

Improving the surface structure of acrylate polymers, for instance, by modifying and incorporating phosphate or carboxyl groups into the structure of PMMA, can result in less accumulation of microorganisms [64,65]. Copolymers obtained in this way have a negatively charged surface, attract positively charged antimicrobial proteins of saliva, and prevent the adsorption and growth of candida while weakening the mechanical properties of acrylates [66]. Given the complexity of denture stomatitis therapy, as well as frequent resistance and relapses, coating and immersion of acrylate restorations in essential oils and extracts can be an alternative in the treatment of denture stomatitis [67].

In addition to the side effects described earlier, the release of free monomers also stimulates oral infection. The release of EGDMA from acrylate polymers stimulates the growth of *Streptococcus sorbinus* and *Streptococcus sangius* in the oral cavity [68].

## 7. Release of Potentially Toxic Substances

After the polymerization procedure, dental acrylates release a certain amount of potentially toxic substances into saliva, where they dissolve and act on the tissues of the mouth and the organism as a whole. These include unpolymerized, unreacted components of the system, as well as secondary products of polymerization. In their concentrated form, these substances are unquestionably very toxic, but their amount dissolved in saliva is very small and depends on the possibility of leaving the structure of the material itself [69].

The biocompatibility of dental materials can be observed in laboratory conditions through their cytotoxicity (viability of cell culture) and cytostaticity (proliferation of cell culture). Literature data show acrylates as mildly to moderately cytotoxic materials [70,71,72,73,74,75]. Discrepancies in the obtained results may be attributed to differences in the experimental design, which include the chosen type of acrylate to be tested, the choice of cell line and the application of different tests for cytotoxicity analysis. In vitro tests do not take into account local and systemic defence reactions of the organism, the flow of saliva and its buffering capacity, and the differences in the composition of natural saliva and the extraction media used. The oral mucosa is more resistant to toxic substances than cell culture, owing to the rough layer that covers its epithelium and large amounts of mucin [76].

## 8. Release of Residual Monomer

Residual monomers (MMA, BuMA, EMA, and UDMA) and crosslinkers (EGDMA, IBMA, etc.) are mostly responsible for the toxic and allergenic effects of acrylates, given that they are not completely polymerized during the bonding of the material [76,77,78,79,80]. Yoshii et al. suggest a greater cytotoxic effect of PBuMA and PEMA compared to PMMA [80]. 

The amount of released monomer is proportional to its total residual amount in the matrix of dental acrylates [32]. Residual monomer leaves the acrylate restoration from its surface parts; therefore, a certain amount remains forever trapped inside its structure [81]. Due to faster wear and a more porous structure, fibre-impregnated acrylates release larger amounts of unpolymerized monomer [82].

According to the standard (ISO 1567:1999), the maximum allowed amount of residual MMA for heat- and cold-polymerized dental acrylates is 2.2% and 4.5%, respectively [11]. Namely, the amount of residual monomer depends on the type of dental acrylates, as well as the method and time of their polymerization. Polymerization at higher temperatures enables a more complete bonding and greater biological value of the material [83]. The polymerization process of cold-polymerized acrylates depends on the chemical initiator, happens quickly, without any pressure, and at a temperature much lower than the *Tg* (glass transition temperature) of acrylate polymers, thus, complete dissolving of the polymer into the monomer is impossible [84]. This results in a more inhomogeneous and porous structure of these materials and a greater possibility of potentially toxic substances and residual monomers being released from them [85,86]. Within the group of heat-polymerized acrylates, a smaller amount of residual monomer and better biological characteristics have been reported. The presence of residual monomer also depends on the thickness of the dental restoration. Consequently, the least amount of residual monomer is found in the palatal part of dentures [87].

To overcome problems regarding allergies to classic acrylates, new, hypoallergenic acrylates are being introduced, with MMA being replaced by diurethane dimethacrylate, polyurethane, polyethylene terephthalate, and polybutylene terephthalate [87]. 

The amount of residual monomer can be reduced by post-polymerization methods (additional heat polymerization inside a cuvette in boiling water, microwave post-polymerization, and immersion of dentures in the water bath before handing over to the patient). Using microwave post-polymerization, the amount of residual monomer can be reduced by up to 25%, which is a consequence of the additional conversion of MMA into polymer, and the evaporation of monomer [86,87,88]. The amount of residual monomer in samples of cold-polymerized acrylates was lowered to a clinically acceptable level with the aforementioned post-polymerization methods [89]. Post-polymerization treatments led to an improvement in the mechanical (crush stress) and biological properties of cold-polymerized acrylates, which can be explained by the reduction of the amount of residual monomer [90]. Regarding heat-polymerized acrylates, post-polymerization methods are not required, given the low values of residual monomer concentration immediately after the polymerization [91]. 

Kostić et al. determined the content of residual monomer methyl methacrylate (MMA) in cold and hot polymerized dental acrylates by using MHE-GC-MS (multiple headspace extraction analysis by gas chromatographymass spectrometry) in combination with GC-FID (gas chromatography with flame-ionization). The amount of residual monomer in cold polymerized acrylate samples was higher (15.75 mgMMA/gPMMA) compared to hot polymerized acrylates (10.96 mgMMA/gPMMA) [92]. 

The findings are in correlation with the opinion accepted in the literature that the largest amount of residual monomer is released into saliva during the first day of using acrylate restorations. The amount of released MMA becomes stable after two weeks of wearing dentures [93,94].

## 9. Other Potentially Toxic Substances

In addition to residual monomer, formaldehyde, benzoyl peroxide, methacrylic and benzoic acid, N,N dimethyl-p-toluidine, hydroquinone, and phthalates may also exhibit a toxic effect on oral tissues due to their release from dental acrylate polymers. Allergic changes can also be caused by inorganic components of dental acrylates, such as cobalt, nickel, and beryllium. An acidic environment leads to the erosion of acrylate dentures and the release of lead and cadmium ions under experimental conditions, with the level of metal release increasing with the increase of temperature [40,95].

Adverse effects of dental acrylate materials in the majority of cases have a local character and disappear rapidly after the removal of the causative agent [96]. They are clinically manifested as cheilitis, stomatitis, burning or scalding sensations in the mouth, painful sensations of varying intensity, as well as candidiasis [97,98]. A more extensive allergic reaction to acrylate dentures in the form of erythema multiforme has also been described [99]. Sensitivity of the mucosa to temporary acrylate restorations is also possible, as well as contact stomatitis in children with removable orthodontic appliances [100,101]. Rare chronic denture stomatitis in the form of fibrous hyperplasia can be seen in elderly patients [102]. On rare occasions, chronic acrylate irritation can also lead to the development of oral cavity carcinoma [103].

Allergies to acrylates belong to group I (anaphylactic reaction) or group IV of delayed hypersensitivity reactions. A total of 1% of the surveyed population is allergic to MMA [104]. Sipahi states that sensitivity to acrylates is exhibited in even 17% of denture wearers (7%) [105]. Local skin contact reactions to BuMA, UDMA, and various crosslinkers have also been clinically described [106]. Not all parts of the mucous membrane of the oral cavity are equally sensitive to acrylate materials, thus, keratinized zones show greater resistance to possible toxic effects [107]. If the mucous membrane is already infected or damaged, it will be more sensitive to the action of acrylate materials [108]. If the allergic reaction is systemic, then it is a generalized dermatological reaction, most often urticaria [95]. 

The potential toxicity of dental acrylates is reduced after immersion into a liquid environment. For these reasons, dentures should be soaked in water at room temperature for 1 to 7 days before handing over to the patient. This especially applies to cold-polymerized dental acrylate restorations, i.e., dentures after repair or underlaying [81,86,93,109,110,111]. Tsuchiya et al. recommend immersing dentures in water at a temperature of 50 to 55 °C for one hour to mobilize free radicals more efficiently and for a more effective subsequent polymerization, especially in the case of a permanent underlay with cold-polymerized dental acrylates [112].

Dental staff and even 20–40% of dental technicians have an allergic reaction to acrylates [113,114,115]. Allergy is most often manifested on the hands, as contact dermatitis with dryness, cracking, peeling of the skin, itching, irritation, and swelling [5,113]. Changes on the skin of the hands are aggravated by mechanical friction, work with plaster, constant temperature changes, and wetting of the hands. Nail diseases are less common [116]. Furthermore, contact dermatitis sometimes appears on the face or eyes of dental personnel [117]. The disease is alleviated at weekends and during annual leave.

Literature data suggest that working with dental acrylates can lead to serious systemic reactions. Moreover, it can incapacitate a person who is in constant contact with the material or even endanger their life [118]. Occupational exposure to acrylate material, monomers, or particles generated during its processing, is also associated with asthma, drowsiness, headache, nausea, anorexia, and decreased gastric motor activity. In addition, it may also lead to neurological disorders, paraesthesia, and neuropathy [118,119].

To protect the personnel when processing acrylate polymers, it is essential to follow the established procedures. Direct contact with unpolymerized mass should be avoided, though even gloves do not always provide adequate protection. Hand hygiene is an important factor in protecting against contact dermatitis. Due to the possibility of inhaling acrylate particles and monomers, it is obligatory to wear a protective mask, as well as to ensure adequate ventilation of the rooms in which the processing takes place [119,120].

Wearing acrylate restorations, primarily dentures, and less often orthodontic appliances, can also cause mechanical damage. It is the result of the contact with the dental restoration, given that acrylate is a hard and non-resilient material [121].

Denture wearers are usually elderly people, which is associated with systemic diseases, malnutrition, the use of numerous medications, changes in the quality and quantity of saliva, and poor oral hygiene [95]. As the flow of saliva is usually reduced in elderly patients, mucosal ulcerations and fungal infections are more common. Therefore, it is key that acrylate dentures lie optimally in the patient’s mouth, are made following the principles of good clinical practice, and that their hygiene is impeccable [122].

The degree of accompanying inflammation is determined by measuring the salivary concentration of proinflammatory cytokines, tumour necrosis factor alpha (TNFα), and myeloperoxidase. A potential allergenic effect is determined by measuring the amount of immunoglobulin E (IgE) in saliva. The results of Kostić et al. showed the proinflammatory but not the allergenic potential of acrylate dentures [123].

It is not possible to precisely determine the values of the minimum number of chemical components of acrylates that could lead to a toxic reaction or tissue sensitization. Given that their harmful effects have already been proven, we should aim for the reduction of their quantity, with strict adherence to the powder–liquid ratio and the polymerization procedure set by the manufacturer.

## 10. Conclusions

Dental acrylate polymers belong to a group of materials widely used in dentistry due to their favourable mechanical, physical and biological properties. Regardless of the satisfactory properties of these polymers that fully meet the requirements of the profession, these materials should be modified and enhanced to avoid any possible harmful effects. Reinforcement of acrylics with fibres and nanofillers improves their mechanical properties, preventing fractures of restorations made from them. Reducing the amount of residual monomer, primarily in cold polymerized acrylates, reduces the frequency of allergic and toxic reactions while improving the physical and mechanical characteristics of these materials. By improving biological properties of dental acrylates with the addition of biocidal agents, infections of oral tissue in contact with dentures are prevented. 

## Figures and Tables

**Table 1:** Powder and liquid composition of conventional acrylate materials.

Liquid (Monomer)	Powder (Polymer)
MMA	PMMA, PEMA, PBuMA
Polymerization inhibitor (0.006% hydroquinone or methoxy phenol)	Polymerization initiator (0.2 to 1.5% dibenzoyl peroxide)
Polymerization crosslinker (EGDMA, TMPT, 1.4-BDMA, 1.6-HDMA)	Plasticizers (dibutyl-, diethyl- and dicyclohexyl phthalate)
Polymerization accelerator (N,N dimethyl-p-toluidine, tertiary amines, sulfuric acid) *	Colours, pigments, and fibres of organic origin that imitate oral tissues and blood vessels

MMA-methyl methacrylate; EGDMA-ethylene glycol dimethacrylate; TMPT- trimethylpropane trimethacrylate; 1.4-BDMA-1.4-butanediol dimethacrylate; 1.6-HDMA-1.6-hexanediol dimethacrylate; PMMA-poly(methyl methacrylate); PEMA-poly(ethyl methacrylate); PBuMA-poly(butyl methacrylate). * Present in cold polymerized acrylates.
